# Planar Electrically Large Structures of Carbon Nanotube Films with High Absorption and Shielding Performance in X-Band

**DOI:** 10.3390/s25133943

**Published:** 2025-06-25

**Authors:** Apostolos Sotiropoulos, Athanasios Masouras, Hristos T. Anastassiu, Vassilis Kostopoulos, Stavros Koulouridis

**Affiliations:** 1 Department of Electrical and Computer Engineering, University of Patras, 26504 Patras, Greece; apssotiro@upatras.gr; 2 Department of Mechanical Engineering and Aeronautics, University of Patras, 26504 Patras, Greece; athmas@upatras.gr (A.M.); kostopoulos@mech.upatras.gr (V.K.); 3 Department of Computer, Informatics and Communications Engineering, International Hellenic University, 62124 Serres, Greece; hristosa@ihu.gr

**Keywords:** electromagnetic compatibility, carbon nanotubes, electromagnetic interference, shielding effectiveness

## Abstract

We consider light, high-absorbance, low-reflectance, electrically large layered sheet structures composed of thin carbon nanotube films. Such structures can be utilized in electromagnetic absorption and shielding applications in the X-band. They are of increasing interest in sensor-enabling technologies, stealth systems, and EMI shielding of electronic components. Especially in aerospace, this is crucial, as sensors are integral to aerospace engineering, enhancing the safety, efficiency, and performance of aircraft and spacecraft. To that end, sheets with carbon nanotube films embedded in a glass fiber polymer matrix are fabricated. The films have a thickness of around 70 μm. As shown, they cause a significant attenuation of the electromagnetic field. For shielding applications, a single-film sheet structure with total thickness of 1.65 mm presents an attenuation of around 25 dB in the transmission coefficient, while the attenuation can reach 37 dB for a two-film sheet structure with thickness of 1.8 mm. Shielding effectiveness performance is found to be greater than 35 dB for the two-film sheet structure. For applications requiring both high shielding and absorption, a two-layered structure with a thickness of 4.65 mm has been designed. The absorption, represented by the Loss Factor, is calculated to achieve values greater than 90%. The simulation results show good agreement with the measured data. The findings demonstrate a promising structure for materials suitable for sensor housings and smart electromagnetic environments where the suppression of electromagnetic interference is critical. In conclusion, the addition of carbon nanotube films, even at micrometer thicknesses, within a glass fiber polymer matrix significantly enhances both electromagnetic shielding and absorption performance.

## 1. Introduction

Significant scientific research is currently under way on the performance of composite materials in electromagnetic compatibility (EMC) applications. Electromagnetic interference (EMI) is a key issue in evaluating electromagnetic compatibility since all electronic equipment and systems should function acceptably in their electromagnetic environment [1].

In aerospace and aeronautical engineering especially, EMI shielding is an increasingly important factor as the electronic components and communication systems become more advanced and densely packed. All modern aircraft structures depend on such systems, which can be highly interfered by electromagnetic fields causing either disrupted communication or more importantly device failures [2]. Such electronic systems are based on sensors that play a critical role in ensuring the safety, efficiency, and reliability of operations. Sensors are integrated into various systems on aircraft, ground control, and air traffic management systems. For example, sensors such as radar altimeters, inertial navigation units (IMUs), GPS receivers, and temperature and pressure transducers are critical for autonomous operation, safety assurance, and real-time system control. The functionality of these sensors is highly vulnerable to electromagnetic interference (EMI), which can degrade signal integrity, introduce measurement errors, or even lead to system-level malfunctions. EMI sources—both internal (e.g., power converters and high-speed switching circuits) and external (e.g., radar systems, jammers, and high-power transmitters)—can disrupt the performance of critical systems such as GPS receivers, radar altimeters, and inertial measurement units (IMUs), potentially resulting in unsafe operational conditions. A recent study [3] highlights the significant impact of EMI on various sensor subsystems within UAV platforms, where compact integration and exposure to harsh electromagnetic environments further exacerbate the issue. Given the safety-critical nature of these sensors, especially in aerospace and autonomous applications, there is a clear and urgent need for effective electromagnetic shielding solutions to mitigate EMI and ensure reliable sensor performance.

Preventing electromagnetic interference and maintaining the integrity of electronic systems can be achieved by incorporating nanocomposite materials, which are well-known for their high shielding characteristics, into aerospace structures [2]. Incorporating such materials into composites or coatings can improve the EMI shielding performance of housings without substantially increasing their weight. Conductive coatings, typically made from metal particles or conductive polymers, offer an extra layer of protection against electromagnetic interference. Various surfaces, including enclosures and components, enhanced with such coatings can provide a flexible and efficient EMI shielding solution. The industry’s pursuit of innovative solutions that balance high performance with EMI interference combining lightweight characteristics determines the trend toward using advanced nanomaterials and conductive coatings for aerospace structures [2].

The applications where nanocomposites for EMI shielding are applicable in aircraft structures vary. Avionics enclosures [4], cable harnesses, radomes enclosing radar systems [5], and antenna housings [6] can be coated with nanocomposite materials, protecting their sensors and limiting the electromagnetic interference. Even composite aircraft structures, including wings and fuselage parts, can be enhanced with polymer matrix nanocomposites in layered or coating forms, providing structural integrity and shielding performance [7,8,9,10]. A category of the above materials for shielding applications are carbon-based nanocomposites such as carbon nanotubes, carbon fibers, and graphene. They are used in various contents because of their high shielding characteristics [11,12,13,14,15,16]. Integrating such materials directly into sensor enclosures, radomes, and supporting structural components offers an effective means to ensure sensor reliability without significantly increasing weight or complexity.

Building on these developments, recent research has demonstrated significant performance enhancements in EMI shielding through the use of advanced carbon-based nanomaterials, especially multi-walled carbon nanotubes (MWCNTs) and emerging 2D materials such as MXenes. For instance, in [17], a robust cellulose nanofiber/MWCNT film with an MWCNT content greater than 50% is developed, with a high level EMI shielding effectiveness (SE), measured using 12 mm samples in a coaxial line setup, underscoring the potential of engineered fibrous composites for lightweight, high-performance shielding applications. MXenes, a family of 2D materials composed of transition metal carbides, nitrides, and carbonitrides, follow a general formula of $M_{n+1}X_{n}T_{x}$, where M represents a transition metal (e.g., Ti, Nb, or V), X is carbon or nitrogen, and T denotes surface terminations, such as hydroxyl, oxygen, or fluorine. Their high surface area, excellent conductivity, hydrophilicity, and chemical stability make them highly effective for EMI shielding. In [18], MXene-based materials—owing to their high conductivity and 2D layered morphology—have been shown to achieve high SE values measured using the waveguide method, making them strong contenders in the domain of ultrathin EMI shielding technologies. In a complementary direction, in [19], a novel measurement setup tailored to evaluate shielding effectiveness across microwave frequencies is developed using a free space approach.

Furthermore, studies on the electromagnetic shielding behavior of nanocarbon-based composites have been presented in [20,21]. In [21], MWCNT/PMMA composites, produced via coagulation techniques, were investigated in the Ka-band (26–37 GHz). Measuring insertion and reflection loss with a scalar network analyzer, it is demonstrated that shielding effectiveness strongly correlates with the MWCNT content and is influenced by the material’s electrical conductivity and dielectric properties. Similarly, in [20], nanocarbon-filled epoxy films are examined using waveguide and resonant cavity techniques, highlighting the roles of the filler type and concentration in determining microwave attenuation. Both studies provided valuable insights into the interaction of electromagnetic waves with CNT-based composites, establishing important principles regarding filler dispersion, percolation behavior, and reflection–absorption mechanisms.

Additionally, a broader review and experimental study [22] has introduced a facile hydrothermal synthesis of flower-like NiO hierarchical structures (rose-flower and silk-flower morphologies) and demonstrated their enhanced dielectric properties and microwave absorption capabilities. Along similar lines, recent developments have emphasized the importance of constructing interconnected conductive carbon networks, such as 3D-printed architectures using graphene and carbon nanotubes, to achieve conformal and high-performance EMI shielding layers. Notably, in [23] a 3D-printed, lightweight carbon-based shielding module that achieved over 60 dB SE is demonstrated while conformally coating electronic components.

Layered structures are also introduced to enhance absorption and shielding applications in [24,25,26,27,28,29]. Absorption and shielding can be increased by controlling the impedance of each layer when matching conditions with the incident electromagnetic radiation are achieved. To do that, we let the electrical properties of each layer vary. Notably, the ratio of the carbon nanocomposites to the material used as the matrix affect the dielectric properties of the latter. By increasing the content of nanocomposite materials in each layer, the electric permittivity and the absorption increase as well. In [24,25,26,27], layered structures based on carbon nanoparticles or graphene are presented. It is shown that even a small increase in the ratio of nanoparticles can highly enhance the performance of the absorption. In [30,31], single wall carbon nanotubes (SWCNTs) in epoxy resin and polyurethane matrices are presented, forming a multilayer design. An incident plane wave is used as excitation of the structure. Absorption, expressed as the Loss Factor (LF %), is examined in the X-band frequency range. It is shown that high absorption percentages can be achieved with such multilayer structures based on carbon nanotube materials [30,31]. Carbon-based nanomaterials embedded in various dielectric and polymer matrices, comprising a range of different materials, are also explored in [32,33,34,35,36]. EMI and shielding performance are highly affected by the content of the polymer matrix. Hence, with the increase in nanoparticles, shielding performance is also increasing [37]. The content of the polymer matrices is based on various materials such as epoxy resin, polyurethane, glass fiber, ethylene vinyl acetate, and polyamide elastomers [38]. However, the mechanical properties of the sample can be affected by overincreasing the loading of the nanomaterials [39,40]. This degradation is primarily attributed to the tendency of nanomaterials to agglomerate at higher concentrations due to strong van der Waals interactions. Such agglomeration results in poor dispersion and the formation of stress concentration sites, which weaken the interfacial bonding between the nanomaterials and the matrix [41].

Dielectric characterization of composite materials is also of great importance for further electromagnetic research. A widely used way to determine the dielectric characteristics of materials is the Transmission/Reflection Line method. A sample is placed in a section of waveguide or coaxial line and the two ports scattering parameters are measured using a Vector Network Analyzer (VNA). The relevant scattering parameters relate closely to the complex permittivity and permeability of the material via mathematical relations. The conversion of S parameters to complex dielectric parameters is implemented using various algorithms, such as the Nicolson–Ross–Weir (NRW) algorithm described in [42,43]. The Transmission/Reflection Line method is commonly used with coaxial lines and waveguides and it has been widely used in the literature [25,26,27,44,45,46,47].

Apart from waveguide measurements, the dielectric characterization of materials can be determined using the free space method. The free space method is a contactless, non-destructive method and allows for measuring larger and planar samples [47]. In addition, it is less prone to measurement errors and requires less material manipulation to carry out the experiment. In that sense, the material tested is much closer to the actual application considered. In the free space method, two antennas are placed facing each other and they are connected to a network analyzer. A sample holder between the two antennas supports the material under test. As seen in [48,49], the minimum transverse dimension of the sample has to be greater than three times the E-plane 3 dB beamwidth of the antenna at the focus point. The free space method can be also used to measure electrical large planar solids [50], materials at a high temperature [51,52], liquids [53], radomes in aerospace [54], multilayered dielectrics [55], composite materials, and metamaterials [56,57,58]. It is also used in several works in millimeter wavelengths for dielectric characterization [59,60,61,62], electromagnetic absorbers, [63] radar-absorbing structures [64,65], shielding effectiveness measurements [66], and nanocomposites [67]. The free space method can be used for measurements in a wide frequency range from a low GHz frequency range (1–6 GHz) [50,52] up to higher frequencies (30–50 GHz) [67].

In this work, we introduce a new experimental and conceptual approach for the evaluation of engineered electromagnetic surfaces based on electrically large area surfaces composed of thin carbon nanotube films fabricated through a relatively simple process and embedded in a glass fiber polymer matrix. Although the constituent CNT films exhibit electrical conductivity, our investigation emphasizes the performance of the composite structures under high-frequency electromagnetic wave exposure applied in the X-band rather than their DC or low-frequency conductive behavior (typically measured in the Hz–kHz range).

Furthermore, the present study introduces a novel synthesis and design approach for carbon nanotube-based structures. Specifically, we propose a simple, scalable, and water-based fabrication method for self-standing multi-walled carbon nanotube (MWCNT) films using polyvinylpyrrolidone (PVP) as a binder. These films are then embedded within a glass fiber polymer matrix to form lightweight, electrically large layered composites. Two configurations are explored: one with a single CNT film at the mid-plane and another with two films placed near the outer layers. This layered integration strategy enables tunable absorption and shielding performance while maintaining mechanical integrity. Unlike many prior works that employ complex hybrid architectures, our approach achieves high shielding effectiveness and absorption using films with micrometer-scale thicknesses.

Previous works [30,31] relied on simulation-based layered designs composed of carbon-based nanomaterials. In [68], waveguide measurements in small-scale samples were carried out in one and two-layer samples. Since the films have a minimal thickness of around 70 μm it is quite difficult to determine their dielectric characteristics. Hence, films are to be embedded in thicker polymer samples, forming layered structures. To this end, layered samples with total thickness of 9.8 mm were fabricated to be measured in a WR-90 waveguide setup.

Here, however, planar samples with thickness around 1.8 mm, much thinner than these required in waveguides in [68], are constructed and measured in the free space with an antenna-based measurement setup in order to examine the absorption and shielding properties of larger surfaces in an electromagnetic environment under realistic, application-oriented conditions. The planar samples in this work are composed of either one or two films for comparison reasons, while in [68], the waveguide samples were constructed with only two films.

The aim of our approach is to design and experimentally validate surfaces with significantly improved absorption and shielding performance. This is enabled not only by the updated measurement methodology but also by a new design philosophy rooted in realistic application constraints. Building on this foundation, the present study introduces thin, lightweight, and scalable shielding structures using carbon nanotube films embedded in composite laminates. These materials are specifically engineered to suppress EMI in environments where sensors are densely integrated, mission-critical, and highly susceptible to electromagnetic disruption. In the simulation phase of this work, we build upon the concepts presented in [30,31,68] and develop novel surface designs grounded in realistic design principles. These surfaces are fabricated and measured, providing full validation and marking a clear step forward from purely simulated or limited-scale studies.

Measurements of S parameters are carried out between 8.2–12.4 GHz (X-band). First, a pair of spot-focusing circular horn lens antennas connected to a Vector Network Analyzer (VNA) are used for the measurements. Spot-focusing antennas are ideal for such measurements since diffraction effects at the edges of the samples can be negligible when spot-focusing lens antennas are used. Still, besides the spot-focusing horn lens antennas, rectangular horn antennas are also used for both comparison and validation reasons. Each measurement setup, either with the lens or the rectangular antennas, requires appropriate calibration, employing time domain techniques. According to the geometric characteristics of the antennas, a simulation setup is designed for the rectangular horn and circular lens antennas. Electromagnetic parameters, such as the electric field, magnetic field, and reflection coefficient, are shown for each antenna used. The radiation pattern is also examined for both lens and horn antennas. Measuring S parameters for the planar structures with both rectangular horn and circular lens antennas verifies that similar results could be achieved when appropriate calibration is applied. After the measurement of S parameters, the dielectric characteristics are determined using the NRW algorithm. Notably, dielectric parameters calculated from S parameters based on horn or lens antenna measurements agree very well. Furthermore, various materials are tested in order to evaluate the effect of carbon nanotube films. The results show that both real and imaginary parts of the dielectric permittivity of the measured samples are highly affected by the carbon nanotube films. In addition, the shielding effectiveness of the measured sheet samples can reach a high level of more than 30 dB isolation due to the use of carbon nanotube films.

To verify the accuracy of the measurements, the free space setup is simulated using the Ansys HFSS (Electronics Desktop 19.2) electromagnetic software tool [69] based on the extracted dielectric parameters of the samples. The simulated and measured results show good agreement, confirming the validity of both the measurement setup and the calculated dielectric parameters.

To explore potential applications where maximum absorption is required, an optimization method is applied to determine the optimal thickness of each layer, aiming to minimize both the S_11_ and S_21_ parameters (i.e., the reflection and transmission coefficients). The robustness of the proposed configuration is demonstrated by varying the layer thickness around the optimal value, with minimal changes observed in performance. The extensive set of results demonstrates that carbon nanotube films, even with micrometer-scale thicknesses, can achieve high absorption and shielding effectiveness, enabling the design of thinner multilayer structures compared to those reported in the literature.

Furthermore, this work contributes both methodologically and practically to the advancement of electromagnetic surface design and validation by enabling the reliable, scalable evaluation of structures designed for real-world use.

## 2. Materials and Methods

### 2.1. Material Descriptions

Samples of glass fiber sheets reinforced with carbon nanotube films were fabricated to examine the EM absorption and shielding characteristics. Sheets of glass fiber without carbon nanotubes were used as reference samples. Sample sheets made of polyethylene were also measured for comparison and validation purposes in measurements as they have known dielectric characteristics.

For the fabrication of the carbon nanotube films, multi-walled carbon nanotube (MWCNT) solid concentrates (pellets, CW2-45) were dispersed in distilled water. The weight percentage of MWCNT pellets was kept below 6 wt%, as per the supplier’s guidelines (ARKEMA). Magnetic stirring was employed to ensure the uniform dispersion of the MWCNT pellets, maintaining a constant temperature of 70 °C throughout the process. Then, ultrasonication was applied to the mixture with a probe sonicator for 1 h and at 80 watts power to ensure the elimination of MWCNT agglomerates. Next, a low content (<5 wt%) of the polymer binder (PVP, polyvinylpyrrolidone solution, K 60, 45% in H_2_O, Sigma Aldrich, Taufkirchen, Germany) was added in the mixture to ensure the self-standing nature of the nano-particle film. The mixture was stirred using a magnetic stirrer until a uniform solution with increased viscosity (ranging from 500 to 1000 mPas) formed. It was then applied to a polymer substrate using a doctor blade set to a height of 1 mm. The cast material was left on the polymer substrate until all the water evaporated under ambient conditions. This process resulted in a high-quality, self-standing film composed of approximately 40 wt% polymer nanoparticles, with a thickness of 60 to 70 μm. The fabrication process described above is relatively simple, especially when compared to materials like MXenes [18], which require a much more complex synthesis procedure. It is preferable also to maintain the film thickness within the range of 50–80 μm, as films thinner than 50 μm are considered extremely fragile and difficult to handle.

**Figure 1:**
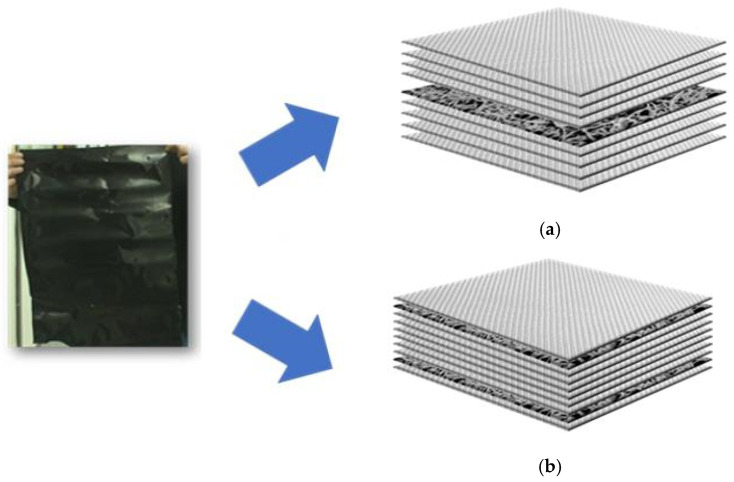
MWCNT/PVP film and integration into a GFRP laminate as the middle layer (**a**) and as interleaves close to the outer layers of the structure (**b**).

In summary, for an aqueous mixture of 500 g, 30 g of MWCNT pellets, 45 g of the PVP solution, and 425 g of distilled water were utilized for the whole process. The MWCNT/PVP films produced were utilized as interleaves in glass epoxy composites. The glass epoxy composite laminates were produced with epoxy-based glass pre-impregnated woven fabrics supplied by SGL Carbon SE (Wiesbaden, Germany). Two configurations were selected, one with a MWCNT/PVT as middle layer of the structure (Figure 1a) [0 deg/90 deg (fiber angle orientation), 4 layers of glass epoxy, 1 layer of MWCNTs/PVP, and 4 layers of glass epoxy] and the second configuration (Figure 1b) was prepared with two layers of MWCNTs/PVP films placed close to the outer layers of the structure [0 deg/90 deg (fiber angle orientation), 1 layer of glass epoxy, 1 layer of MWCNTs/PVP, 6 layers of glass epoxy, 1 layer of MWCNTs/PVP, and 1 layer of glass epoxy]. The whole assembly in both cases was placed in a vacuum bag and the whole structure was placed in an autoclave for the curing process. The curing process of the composite structure was conducted at a 140 °C temperature and 4 bars of pressure for 2 h.

**Figure 2:**
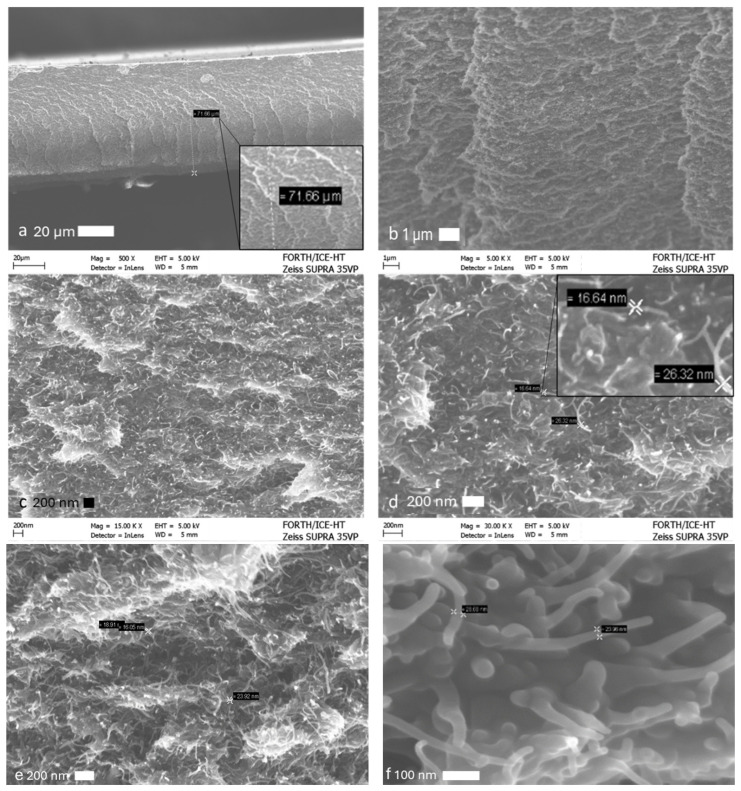
(**a**) Cross-sectional area of the film, with a thickness measured at ~70 μm (magnification: 500×). (**b**) MWCNT network in the film structure (5000×). (**c**) Closer view of the MWCNT network (25,000×). (**d**) MWCNT diameter measurement (30,000×). (**e**) Closer view of the internal structure of the MWCNT/PVP film and diameter measurement (30,000×). (**f**) Very close view of the MWCNTs in the structure of the film (~100,000×). Reproduced with permission from [68].

An investigation of two different structures was carried out to evaluate and compare the influence of the structural configuration on the electromagnetic absorption and shielding performance. Each structure embodied a distinct approach in terms of material layering and geometric arrangement. By analyzing both designs, we aim to better understand the relationship between structural architecture and performance characteristics, particularly how variations in design affect the dielectric properties, absorption efficiency, and overall shielding effectiveness. SEM micrographs of the materials are shown in Figure 2a–f. The uniform dispersion of nanoparticles within the microstructure of the epoxy nanocomposites has been confirmed.

The surface electrical conductivity of the freestanding MWCNT/PVP films used in this study was measured to be approximately 500 S/m using the standard four-point probe method. Measurements were conducted in the low-frequency range (<100 Hz), which is ideal for minimizing the influence of inductive and capacitive effects and ensuring accurate resistance characterization. A KEITHLEY DMM 2002 multimeter (Keithley, Cleveland, OH, USA) was used, and the measured resistance was converted to sheet resistance and subsequently to specific and surface conductivity. While this value reflects the film’s inherent electrical properties at very low frequencies, it is important to note that our study focuses on the GHz-range (X-band), where electromagnetic behavior is dominated by dielectric loss, impedance matching and wave–material interactions rather than static conductivity. Therefore, high-frequency performance is more accurately captured through complex permittivity and S parameter measurements.

**Figure 3:**
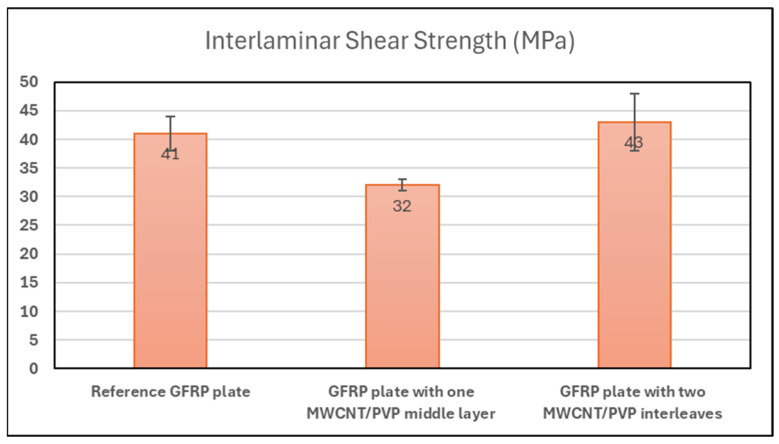
Average interlaminar shear strength of the modified GFRP plates with one and two layers of MWCNT/PVP films at the outer layers of the composite.

Furthermore, to evaluate the composite’s mechanical integrity under interlaminar shear stress, short beam shear tests were conducted in accordance with ASTM Standard D2344. Flat specimens with dimensions of 10 mm in length and 5 mm in width were subjected to a three-point bending load configuration. Figure 3 presents the average interlaminar shear strength (ILSS) for glass fiber-reinforced polymer (GFRP) composites modified with one or two layers of MWCNT/PVP films positioned near the outer surfaces of the laminate. The results indicate that specimens with two interleaved MWCNT/PVP films exhibit negligible change in the ILSS compared to the unmodified reference. Notably, when a single MWCNT/PVP layer is placed at the mid-plane, a slight reduction in the ILSS is observed. However, placing the films near the outer surfaces either maintains or slightly enhances the shear strength. This suggests that the mechanical properties of the composite are preserved—or potentially improved—by the strategic placement of the MWCNT/PVP films, thus ensuring structural integrity alongside enhanced electromagnetic functionality.

**Figure 4:**
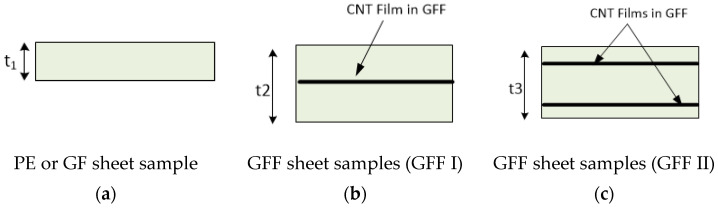
Structural description of the samples of the materials.

Carbon nanotube films have a thickness on the order of μm, while the total thickness of the final sheet is on the order of mm. Sheets of glass fiber without carbon nanotubes films are referred to as GF (glass fiber), while the sheets of glass fiber reinforced with carbon nanotubes films are referred to as GFF (glass film fiber). The GFF sheet samples are composed of carbon nanotubes films placed intermediately in the glass fiber polymer matrix, forming a layered structure. Two types of GFF samples have been manufactured. The first type sample consists of a single carbon nanotube film denoted as GFF I and is shown in Figure 4b, while the second, denoted as GFF II, has two embedded films and is shown in Figure 4c.

More than one sheet samples (either two or three) of both GF and GFF were fabricated in order to minimize random errors during the fabrication process. GF sheets have a thickness of t_1_ = 1.55 mm, the GFF I thickness is t_2_ = 1.65 mm, and GFF II is t_3_ = 1.8 mm thick. The side dimensions of the samples are 21 cm × 21 cm, corresponding to 5.7 λ up to 8.7 λ for the X-band frequency (8.2–12.4 GHz), justifying their classification as electrically large structures for the purposes of the electromagnetic analysis and shielding performance evaluation. All types of samples are analytically presented in Table 1. They were constructed in the Department of Mechanical Engineering and Aeronautics of the University of Patras.

### 2.2. Absorption—Shielding Effectiveness

The electromagnetic absorption of an incident EM wave by a material is typically associated with the skin depth, as discussed in [68]. The attenuation of EM waves and its connection to electromagnetic shielding are also analytically presented in [68] through mathematical formulations. Shielding effectiveness (SE), a key measure of electromagnetic shielding, can be expressed as the sum of three components: reflection, absorption, and multiple internal reflections.

**Figure 5:**
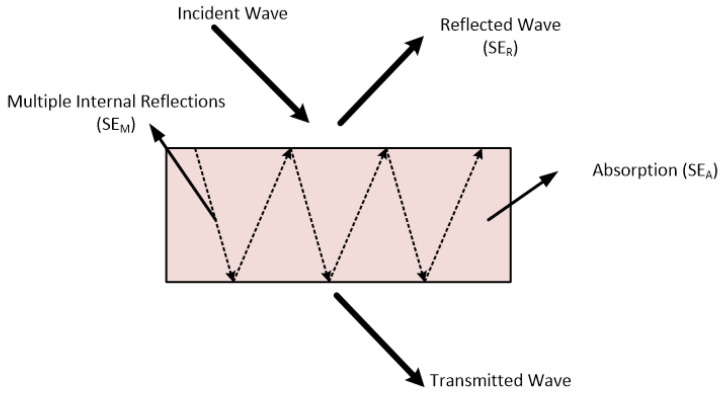
Description of shielding terms of an incident wave. SE_R_ is for reflection, SE_A_ is for absorption, and SE_M_ is for multiple internal reflections.

In Figure 5, an incident EM wave is shown, illustrating these three terms. The following equation expresses shielding effectiveness according to reflection, absorption and multiple internal reflections:

(1) $${SE}_{T}={SE}_{R}+{SE}_{A}+{SE}_{M}$$

${SE}_{R}\;$ refers to the reflection term, ${SE}_{A}\;$ refers to the absorption term, and ${SE}_{M}\;$ refers to multiple internal reflections inside the material.

${SE}_{M}$ can be neglected when ${SE}_{A}$ in Equation (1) is more than 15 dB [11,39,70,71]. Skin depth also influences the effect of multiple internal reflections within the shielding material. When the shield’s thickness is less than the skin depth, these internal reflections reduce the overall shielding effectiveness. Conversely, if the shield is thicker than the skin depth, the contribution of multiple internal reflections becomes negligible [70]. Therefore, in such cases, shielding effectiveness is primarily governed by absorption and reflection.

Shielding effectiveness can be also determined from measured or calculated scattering parameters through the mathematical analysis described in [11,45,68,70,72,73,74]. The absorption, expressed as the Loss Factor (LF %), can be expressed by the following equation [68]:

(2) $$LF\left(\%\right)=\left(1-{\left|S_{11}\right|}^{2}-{\left|S_{21}\right|}^{2}\right)100\%$$

For the maximum absorption of the structure, both S_11_ and S_21_ have to be minimized.

The electromagnetic absorption of an EM wave through a material is also highly dependent on its intrinsic wave impedance (η), calculated as

(3) η=με 

where ε and μ are the complex electric permittivity and magnetic permeability, respectively.

The intrinsic wave impedance also influences the reflection at the interface between an infinite material and free space. The reflection coefficient can be calculated using the following equation, as presented in [44]:

(4) $$RC=\frac{\eta_{M}-\eta_{0}}{\eta_{M}+\eta_{0}}$$

where $\eta_{0}\;$ is the free space impedance, $\eta_{0}=377\;$ Ohms, and $\eta_{M}\;$ is the material impedance. For the minimum reflection coefficient, the impedance material $\eta_{M}$ must be close to the impedance of the free space, establishing a matching condition.

Based on the above analysis, electromagnetic shielding is strongly associated with minimizing transmission and maximizing attenuation of the EM field through a material. The transmission coefficient, denoted as S_21_, represents the ratio of the transmitted wave to the incident wave. A lower S_21_ value indicates greater attenuation, and consequently, improved EMI shielding performance. Absorption loss—generally proportional to the shield’s thickness—is the dominant factor determining the total shielding effectiveness [70]. While shielding effectiveness primarily depends on the S_21_ parameter, achieving high absorption also requires a low reflection coefficient [68], as indicated by Equation (2), which shows that the absorption Loss Factor is influenced by both reflection and transmission. As shown in Equation (4), achieving a low reflection coefficient necessitates impedance matching between the material and free space.

It is important to note that while the primary absorption mechanisms in the present composite structures are mainly attributed to dielectric losses, there remains the potential for localized thermal effects, particularly due to Joule heating associated with induced currents in conductive nanostructures. Multi-walled carbon nanotubes (MWCNTs) can exhibit resistive heating when electromagnetic energy induces current flow through percolated or partially connected pathways. This phenomenon has been documented in various CNT-based systems. For instance, CNT/polyurethane composites have shown temperature rises of several tens of degrees Celsius at relatively low voltages [75], while CNT/waterborne polyurethane films have reached surface temperatures of approximately 77 °C under a 20 V stimulus [76]. Additionally, electrical bias across individual MWCNTs has resulted in localized heating of 5–42 K per microwatt of dissipated power, indicating their sensitivity to Joule heating effects [77].

In our study, however, the MWCNTs are embedded in a non-conductive polyvinylpyrrolidone (PVP) matrix and dispersed in such a way that continuous conductive paths are avoided, limiting the likelihood of significant macroscopic thermal effects. Under the low power levels and short exposure durations used in this work, no measurable thermal degradation, material instability, or deviation in electromagnetic performance was observed during repeated tests. The films remained physically intact and functionally stable, indicating effective energy dissipation mechanisms dominated by dielectric losses rather than heat accumulation.

## 3. Experimental Setup and Results

### 3.1. Measurement Setup

**Figure 6:**
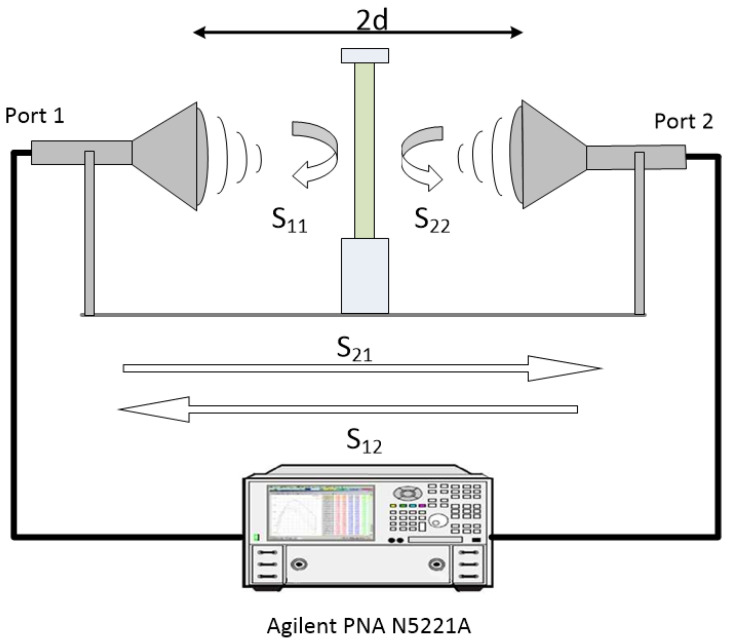

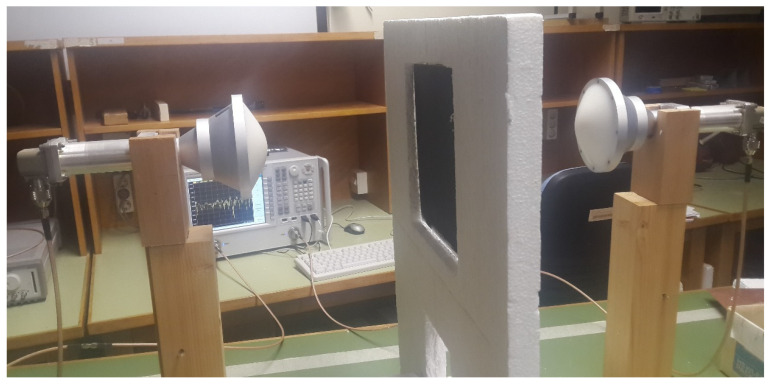
Schematic illustration of the free space measurement setup using spot-focusing horn lens antennas.

The determination of the electromagnetic properties and the dielectric characterization of the materials require the measurement of scattering parameters (S parameters). For measuring the S parameters in free space, two identical antennas are used. The measurement system is shown in Figure 6. The setup consists of a two-port Vector Network Analyzer (VNA) PNA N5221A (Keysight, Santa Rosa, CA, USA) (13 MHz–13.5 GHz), a pair of horn antennas, and a sample holder hosting the materials under test (MUT) made of styrofoam. Three types of horn antennas were used for comparison and verification. First, a pair of spot-focusing horn lens antennas was used. Their model was SAQ-103039-90-S1 [78] and they operate in the X-band frequencies with a center operating frequency of 10.3 GHz. They deliver a 10 dB spot size of 1.75″ at a focal length of 3.94″ (10 cm), according to the datasheet [78]. The second pair was rectangular horn antennas with a x b aperture (a = b = 76 mm) for X-band frequencies. Finally, a pair of two Aaronia powerlog 40400 (Aaronia AG, Strickscheid, Germany) [79] rectangular horn antennas with smaller c × d aperture (c = 38 mm, d = 55 mm) were also used for the measurements.

Measurements were carried out at a distance of d = 10 cm (see Figure 6) for the lens and Powerlog 40400, while for X-band rectangular horn antennas, measurements were carried out at distances of d = 10 cm and d = 20 cm. The measurements were performed at the specified distances to enhance the reliability and repeatability of the experimental results. Conducting measurements at multiple distances allows for the verification of the consistency of the electromagnetic response, ensuring that the measured S parameters used for the calculation of the absorption and shielding characteristics are not influenced by distance-related artifacts or setup-specific factors. Additionally, repeating measurements at different positions helps reduce the impacts of random environmental fluctuations, measurement noise, and alignment inaccuracies. This approach improves the statistical robustness of the data and enhances the overall measurement accuracy.

The experimental setup was configured with a VNA output power of 5 dBm and 841 frequency steps, corresponding to a frequency resolution of 5 MHz. In this setup, S_11_ and S_22_ represent the reflection coefficients at port 1 and port 2, respectively, while S_21_ and S_12_ correspond to the transmission parameters from port 1 to port 2 and from port 2 to port 1, respectively.

### 3.2. Calibration

In free space measurements with horn antennas, multiple reflections between the antennas and the mode transitions via the surface of the sample are expected. As a result, S parameter measurement errors can arise. In order to set the reference planes close to the surface of the examined sample and remove the effects of multiple reflections, calibration has to be firstly applied. A calibration method namely GRL (Gated–Reflect–Line) is carried out according to [11,80], considering also time gating parameters. It was performed to ensure the accurate de-embedding of the sample’s response from the measurement system. Time gating also eliminates the diffraction of energy from the edge of the antennas. It is also used to ensure that there are not multiple reflections from the sample itself.

The GRL calibration is in the TRL (Transmission Reflect Line) family of calibration methods and is similar to LRM (Line–Reflect–Match) calibration procedure. In these calibration methods, three standards are used to ‘model’ the perfect conditions for the VNA. For the line standard, the sample holder is left empty, which is similar to a through standard. This establishes a baseline with 100% transmission (no material present) through the air into the opposite receiver antenna, effectively serving as a through calibration. For the reflect standard, a metal plate of a known thickness is used to reflect all the incident energy back to the source (transmitter). With a metal plate, both ports are calibrated with a single standard. This defines the reference for maximum reflection. The metal plate is also used as an isolation standard, as no signal should be detected at the opposite antenna. Finally, the matching standard corresponds to an ideal load, where all transmitted energy is absorbed and no reflections are measured at the transmitter. Under such conditions, the receiver would detect an electromagnetic signal ideally below the noise floor. In theory, this could be achieved by directing the transmitting antenna upward into free space (e.g., the open sky), where no reflective surfaces are present. However, as this is rarely practical in laboratory environments, the matched condition is instead approximated using time-domain gating techniques. In our setup, reflections from the metal plate (used as the reflect standard) are time-gated to isolate and remove undesired echoes. This process effectively simulates a perfect matching condition—i.e., no reflection and no transmission—without requiring a physical absorber with ideal characteristics.

To begin with, a full two-port calibration must be performed up to the coaxial-to-waveguide adapter using a waveguide calibration kit. A SOLT (Short, Open, Load, Thru) calibration is performed at the end of the cables.

**Figure 7:**
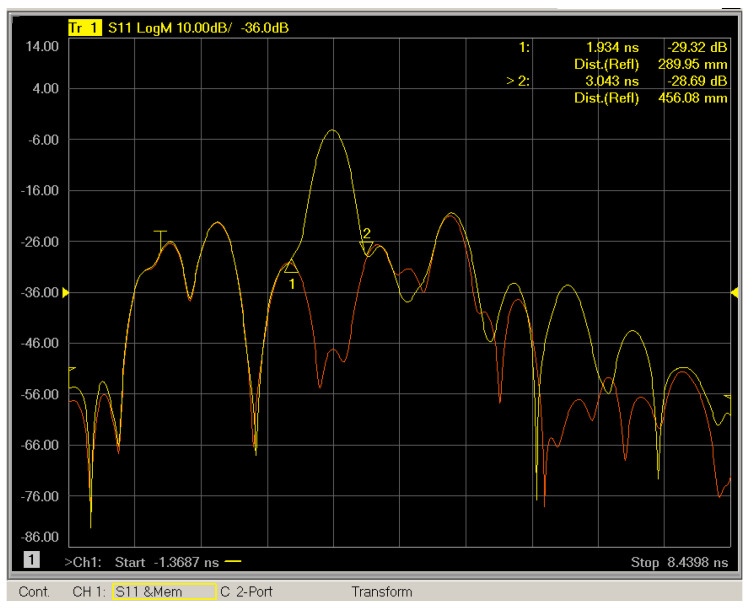
S_11_ measurement in the time domain with (yellow line) and without (red line) metal plate for calibration purposes as has been captured from Vector Network Analyzer screen during calibration process. Markers 1 and 2 are making the window of interest for which time gating will be applied as part of the process.

Afterwards, to carry out measurements at the surface of the sample, the reference planes should be transferred from the end of the cables to the surface of the sample. Hence, the position of the metal plate and the length of the fixture must be determined. For this reason, measurements of the reflection coefficient in time domain were carried out with and without the metal plate. In Figure 7, the S_11_ response in time domain is depicted for both air and metal plate measurements. The red line refers to the air measurement without the metal plate while the yellow line shows the reflection in the case of the metal plate measurement. The time between marker 1 and marker 2 determines the position of the metal plate. Consequently, time gating parameters are determined. Marker 1 points at the start time and marker 2 is on the stop time. This time domain technique is repeated for all the antenna pairs used for calibration purposes. When the measurement distance changes, the calibration process is repeated. It is noted that for the best accuracy results, the thickness of the metal plate should be equal to the thickness of the sample. By applying the above approach, the GRL calibration ensures accurate reference plane positioning and reliable extraction of material-specific S parameters.

Although the antennas used in free space measurements are the same, they will be hardly identical. Electrical differences between the two antennas can occur due to some imperfections during the fabrication process. To overcome these problems, reflection coefficients of both antennas in time domain (S_11_ and S_22_) can be used. Only after adjusting the physical distance of the antennas, so that the curves of both reflection coefficients S_11_ and S_22_ in time domain match, will the electrical distance of the antennas be the same. In this way, GRL calibration will achieve the best accuracy [80]. After the above calibration of the VNA, the S parameters of an empty sample holder without any material are measured by placing the sample holder midway between the two antennas. In this way, measurements of the free space are carried out in order to use them as a reference later on. Afterwards, each material under test (MUT) is placed in the sample holder fixture between the antennas, and S parameter measurements are carried out.

### 3.3. Antenna Modelling and Simulation

#### 3.3.1. Antenna Modelling

**Figure 8:**
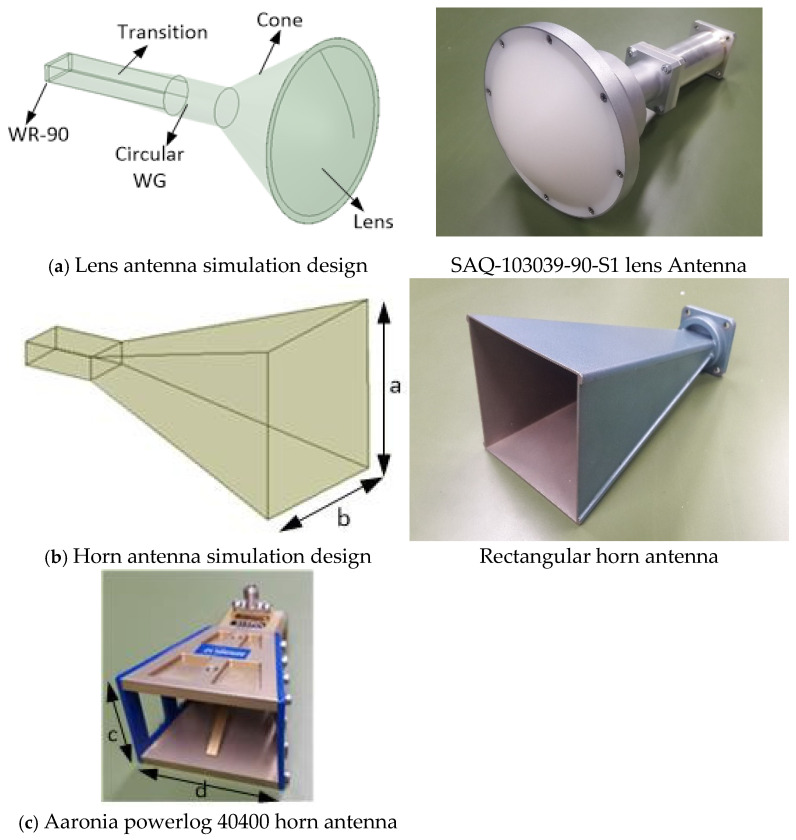
Lens and horn antenna designs.

The measurements were carried out with three types of horn antennas, a lens horn antenna shown in Figure 8a, a rectangular horn antenna shown in Figure 8b, and a smaller horn antenna shown in Figure 8c, as described in Section 3.1. The lens antenna is composed of five parts. Firstly, a WR-90 waveguide type (cross-sectional dimensions 22.86 mm × 10.16 mm) is used to connect the antenna with the waveguide to coaxial adapter. Then, a transition from rectangular to circular waveguide is used. The circular waveguide is connected to a cone horn and finally a dielectric lens is placed at the end. The dielectric lens is used in order to focus the EM waves to a specific distance. While the lens antenna can focus EM radiation at a specific distance, rectangular horn antennas have a wider radiation pattern. However, with appropriate calibration and using time-domain techniques, both types of antennas could measure the S parameters referenced to a specific point if distance is kept relatively short.

#### 3.3.2. Antenna Simulation

**Figure 9:**
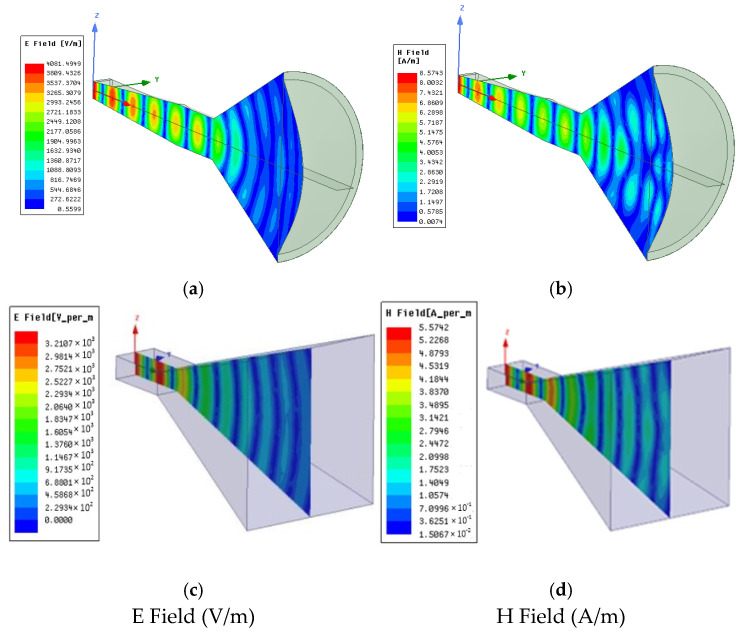
Electric fields (**a**,**c**) and magnetic fields (**b**,**d**) for the lens and horn antennas used for the measurements.

A simulation of the antenna measurement system is usually necessary for further investigation purposes. The lens antenna (Figure 8a) and rectangular horn antenna (Figure 8b) are simulated with the Ansys HFSS electromagnetic simulation software tool [69] in order to compare their characteristics. The lens antenna is designed according to the step file in [78] (see Figure 8a). A WR-90 waveguide port (cross-sectional dimensions 22.86 mm × 10.16 mm) is used for its excitation. A rectangular to circular waveguide transition is used in order to connect the rectangular to the circular waveguide, which then feeds the circular cone. The circular cone is loaded with a dielectric lens with a relative permittivity ε_r_ = 2.35 and loss tangent tanδ = 0 for simulation purposes. PEC boundary conditions have been assigned to all the metallic surfaces of the antenna. In Figure 9a,b, a plot is shown for both the electric and magnetic fields propagating through the lens antenna, while in Figure 9c,d, the respective field plots are shown for the rectangular horn antenna after its simulation with the electromagnetic software.

**Figure 10:**
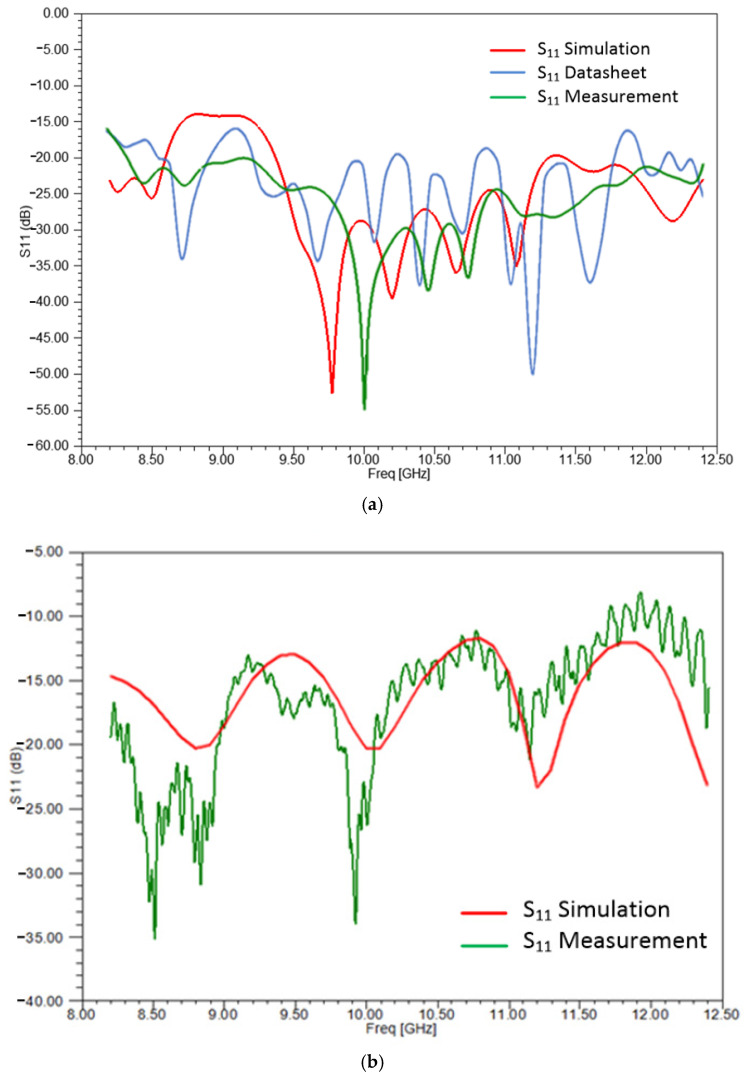
(**a**) Comparison of the S_11_ for the spot-focusing lens antenna among the simulation, datasheet, and measurements. (**b**) Comparison of the S_11_ for the rectangular horn antenna between the simulation and measurements.

In Figure 10a, a comparison for the reflection coefficient, S_11_ (dB), of the lens antenna is carried out. As seen, there is satisfactory agreement among the S_11_ of the lens antenna from the datasheet of the manufacturer (blue line) [78], the S_11_ extracted through the measurement in the laboratory (green line), and the S_11_ computed by the simulated design of the antenna (red line).

In Figure 10b, a comparison of the reflection coefficient, S_11_ (dB), is shown for the rectangular horn antenna. The results are in satisfactory agreement between the simulation (red line) and measured data (green line). For both figures above, the small deviations between the simulated and measured data can be attributed to the assumptions inherent in the simulation model, which considers idealized conditions such as perfect material uniformity and ideal boundary conditions. In contrast, the actual system—especially in free space measurements—is influenced by real-world factors such as fabrication tolerances, surface roughness, minor structural asymmetries, and environmental conditions present in the laboratory setting. These non-idealities can significantly influence the measurement process, especially at lower signal levels, where their impacts become more pronounced.

**Figure 11:**
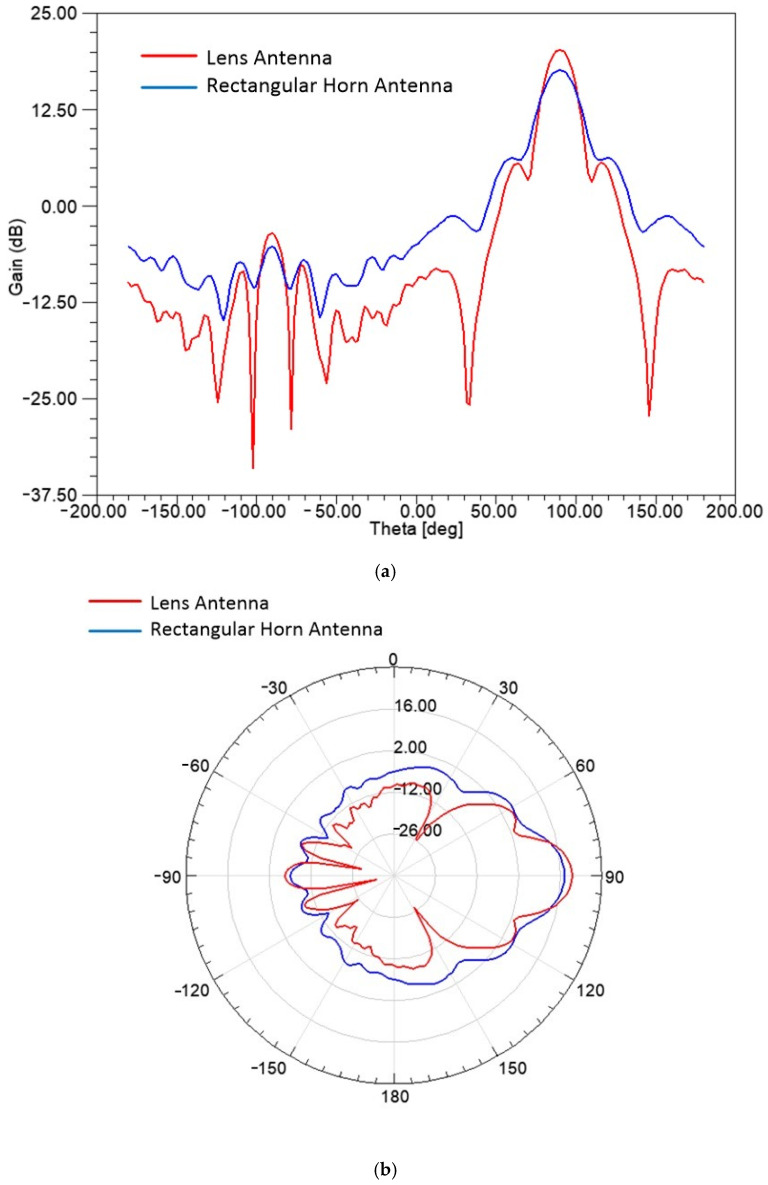
Gain plots for the horn and lens antennas in Cartesian (**a**) and polar (**b**) plots for f = 10 GHz.

In Figure 11, the realized gain patterns of the rectangular horn and lens antennas, as computed by the simulated design, are presented for f = 10 GHz, in Cartesian (Figure 11a) and polar formats (Figure 11b). As seen, both antennas have similar radiation patterns, and they can be used for measurements of the sheet samples after calibration.

### 3.4. Dielectric Characterization

Following the measurement of the S parameters, the dielectric properties of each sample sheet were calculated using the Nicholson–Ross–Weir (NRW) algorithm [42,43], a widely used method for determining the dielectric parameters of materials based on the S parameters [25,27,44,81]. For the determination of the dielectric parameters of the materials, the S parameters of the three pairs of antennas described is Section 3.3.1 were used for comparison and verification.

**Figure 12:**
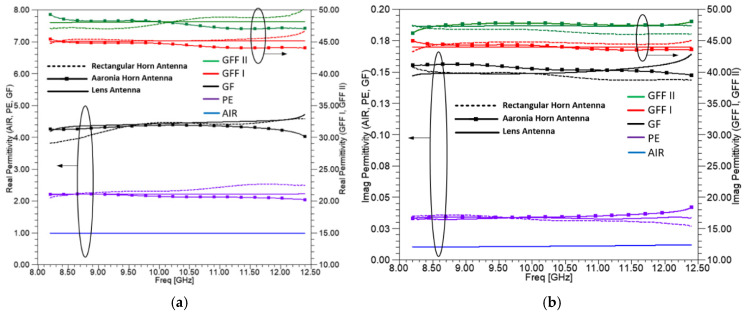

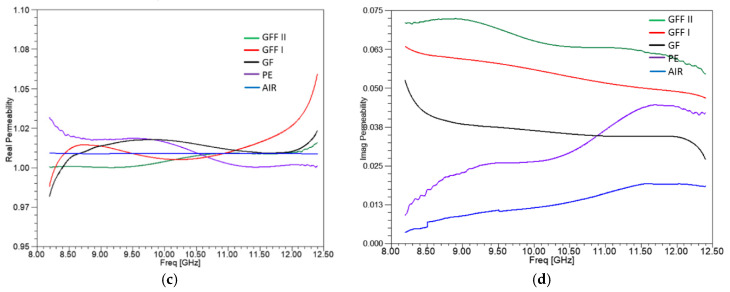
Real (**a**,**c**) and imaginary (**b**,**d**) parts for the materials measured (Air, PE, GF, GFF I, GFF II). For ε′ and ε″ (**a**,**b**), the results are shown as mean values for horn and lens antennas. For μ′ and μ″ (**c**,**d**), the results are shown as mean values only for lens antennas.

Figure 12 presents the calculated real and imaginary components of electric permittivity (ε′, ε″) in panels (a) and (b), and magnetic permeability (μ′, μ″) in panels (c) and (d), plotted as functions of frequency for each measured sample. Each plot displays the mean values obtained across all tested samples and for each antenna pair used during the measurements. Free space results are also shown for reference purposes. Magnetic characteristics (real and imaginary parts of magnetic permeability) are shown only for the lens horn antennas, since the samples have no magnetic properties and the results are quite identical for all the types of antennas used.

As shown in Figure 12, both the real and imaginary parts of relative permittivity have similar values for the same material independent of the type of the antenna used. This is of course expected. Still, it is based on the meticulous calibration procedure. Since the dielectric parameters are closely related to the S parameter measurements and the reference plane has been placed at the surface of the material, after successful calibration, the S parameters should be similar. From a different point of view, this is also a validation of correct calibration.

Polyethylene (PE) samples have a real part of relative permittivity around 2.2, which is validated by the dielectric constant found in the literature. GF samples have a real part of relative permittivity around 4.4, verified also by measurements with the waveguide method in [68]. However, GFF I and GFF II materials have much higher values, around 50, as shown in right axis of Figure 12a. As for the imaginary part of relative permittivity, similar results are noticed for PE and GF. Specifically, the imaginary part of relative permittivity ranges from 0.03 to 0.15. On the contrary, for the GFF I and GFF II materials, it ranges from 44 up to 48, as shown on the right axis of Figure 12b. The high permittivity observed in GFF materials can be attributed to their layered architecture, composed of successive layers of carbon nanotube films. It is important to note that the real part of the complex permittivity corresponds to the energy stored due to polarization, while the imaginary part is associated with dielectric losses or energy dissipation [82,83].

**Figure 13:**
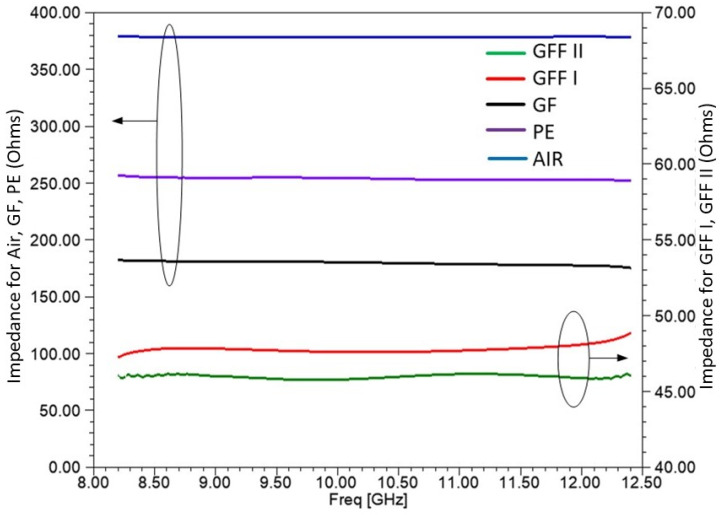
Intrinsic wave impedance n for the materials measured. Air, PE, and GF are shown on the left axis, and GFF I and GFF II are shown on the right axis.

In Figure 13, the magnitude of the wave impedance η calculated with Equation (3) is shown for each material measured in the frequency range of 8.2–12.4 GHz. The magnitude of free space ($\eta_{0}=377\;$ Ohm) is also shown for reference purposes. As shown in the right axis, both GFF I and GFF II present a much lower impedance around 50 Ohms, while the impedance of GF and PE is around 200–250 Ohms. Consequently, a high reflection coefficient of an incident plane wave onto structures based on GFF I or GFF II materials is expected due to the significant impedance mismatching, according to Equation (4).

**Figure 14:**
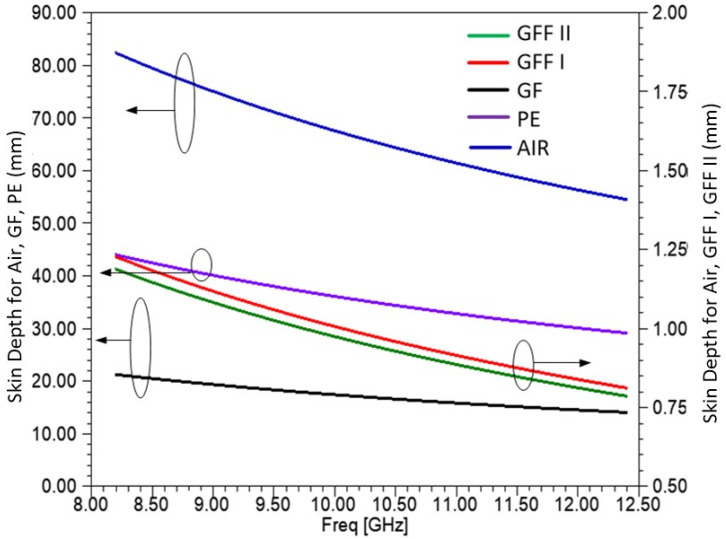
Skin depth calculation in mm for the materials measured. Air, PE, and GF are shown on the left axis, and GFF I and GFF II are shown on the right axis.

In Figure 14, the skin depth is shown for each material measured over the X-band frequencies. The skin depth of the GF sample, shown on the left axis, was also compared with the corresponding one in [68]. It is found that its value is of the same order. As shown, the skin depth of PE and GF is around 20–40 mm. On the contrary, the skin depth of both GFF I and GFF II materials, shown on the right axis, is around 1 mm, depicting the high attenuation of an incident EM wave. Similar results have been found for GFF materials measured with the waveguide method in [68].

### 3.5. Measurement Validation

After determining the dielectric properties of the materials, free space measurements are carried out according to the setup shown in Figure 6 in order to validate the simulated S parameters based on the calculated dielectric parameters over the measured S parameters. This will validate the dielectric characterization experimental setup process. The S parameters of samples of polyethylene (PE), GF, GFF I, and GFF II were measured. Measurements were carried out with the rectangular horn antennas at a distance of d = 20 cm from the sample sheet after successful calibration and determining time domain parameters according to Figure 7.

A simulation setup was also designed in the electromagnetic software HFSS [69]. Rectangular horn antennas were used in the simulation setup. Each antenna was excited by a waveport [69] to simulate the measurement setup. Each material/sample was simulated using the dielectric properties obtained in Section 3.4.

Since the electromagnetic simulations in this study were conducted in the X-band frequency range, where the corresponding free space wavelength is approximately 3 cm, the nanoscale structural features of the CNT/polymer composite—such as the multi-walled carbon nanotube (MWCNT) diameter (~10 nm) and interparticle spacing—are several orders of magnitude smaller than the wavelength. As a result, these fine structural details cannot be explicitly resolved at the mesh resolution typically used in full-wave solvers. Yet such details cannot affect the macroscopic field distribution at the frequencies under investigation. Consequently, we adopted a homogenization-based effective medium approach, wherein the CNT/PVP composite layers are modeled as lossy dielectric slabs characterized by bulk-equivalent dielectric characteristics. These effective electromagnetic properties are extracted directly from experimental measurements. This modeling strategy is well-established in the electromagnetic shielding and absorption community for analyzing structurally complex, subwavelength-scale composites [84]. In this framework, we do not attempt to replicate the internal nanostructure of the CNT network; instead, we focus on capturing the bulk electromagnetic behavior of the material at the macroscale. This allows for accurate evaluations of the field distributions, reflection and absorption characteristics, and interactions within multilayered planar structures—consistent with practical device-level implementations and experimental configurations.

**Figure 15:**
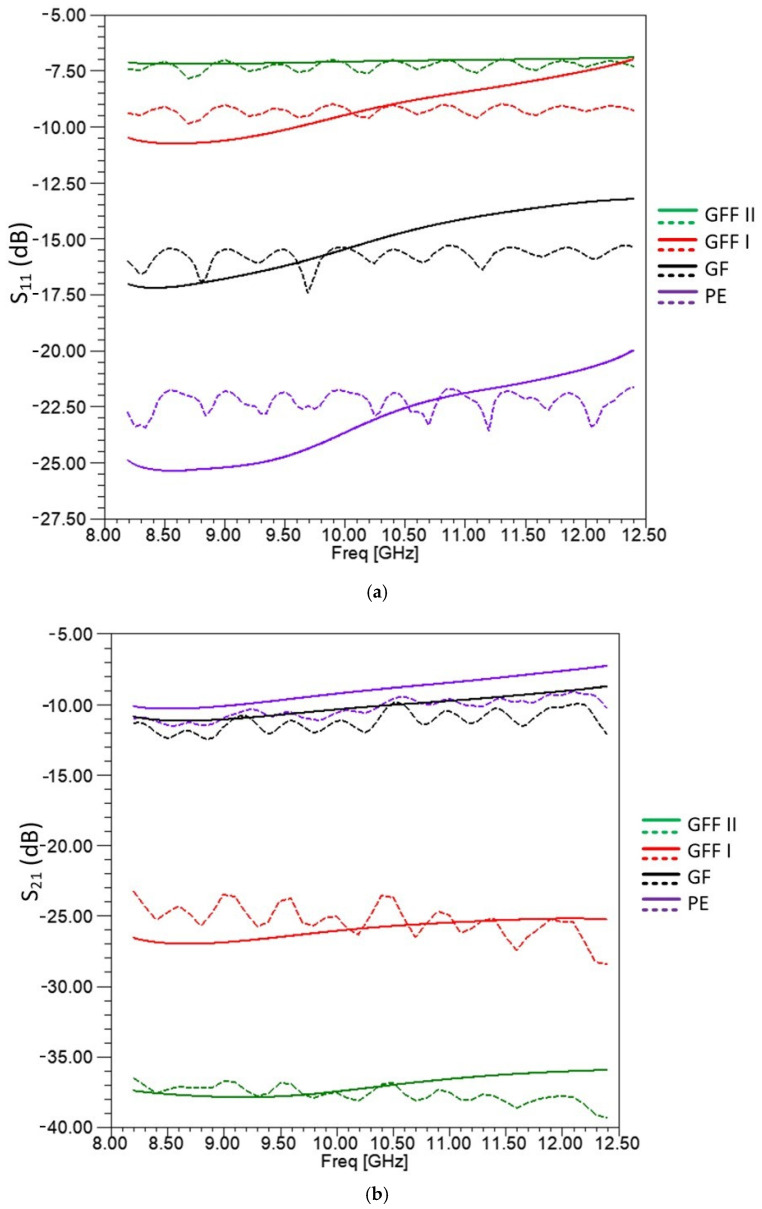
(**a**) S_11_ (dB) between measured and simulated results for the samples of the materials (PE, GF, GFF I, and GFF II). The dashed lines refer to the simulation and the solid refers to measurements. (**b**) S_21_ (dB) between measured and simulated results for the samples of the materials (PE, GF, GFF I, and GFF II). The dashed lines refer to the simulation and the solid refers to measurements.

The calculated and measured S parameters are presented in Figure 15a, b. As shown in the S_11_ and S_21_ plots, the simulated results with average dielectric constants demonstrate satisfactory agreement with the experimental measurements, thereby validating the measurement setup and procedure.

As is shown in Figure 15a,b, in comparison with other samples, GFF I and GFF II have a slightly higher S_11_ while they have a significantly lower S_21_. In particular, S_21_ for GFF I sheet samples is around −15 dB lower than PE and GF samples, while S_21_ for GFF II samples is around −25 dB lower. Indeed, as seen, GFF II samples have an S_21_ transmission coefficient from −35 dB up to −41 dB, while GFF I samples have an S_21_ transmission coefficient of around −26 dB. GFF sheet samples present an S_11_ parameter from −7.5 to −10 dB, showing a quite high reflection performance. Notably, the measured S parameters of the GF and GFF samples with the free space method present also a similar performance with the measurements presented in [68], where waveguide method was used. In [68], carbon nanotube films were examined, forming two-layered waveguide samples. These samples consisted of a thick epoxy resin layer combined with either multiple layers of glass fibers (referred to as Resin-GF) or an alternating structure of glass fiber layers interchanging with carbon nanotube films (referred to as Resin-GFF). As reported in [68], the Resin-GFF samples, enhanced with carbon nanotube films, exhibited a transmission coefficient below −20 dB. The reflection coefficient of Resin-GFF samples in [68] had values from −10 dB up to −5 dB around X-band frequencies, since the sample in the waveguide had two layers, an epoxy layer followed by the GFF layer.

As a result, by comparing the measurements of the S parameters for the GF and GFF samples in [68] and in this work, it is clearly assumed that carbon nanotube films placed inside the GFF sheet can greatly affect the transmission coefficient of an EM wave.

### 3.6. Shielding Effectiveness

**Figure 16:**
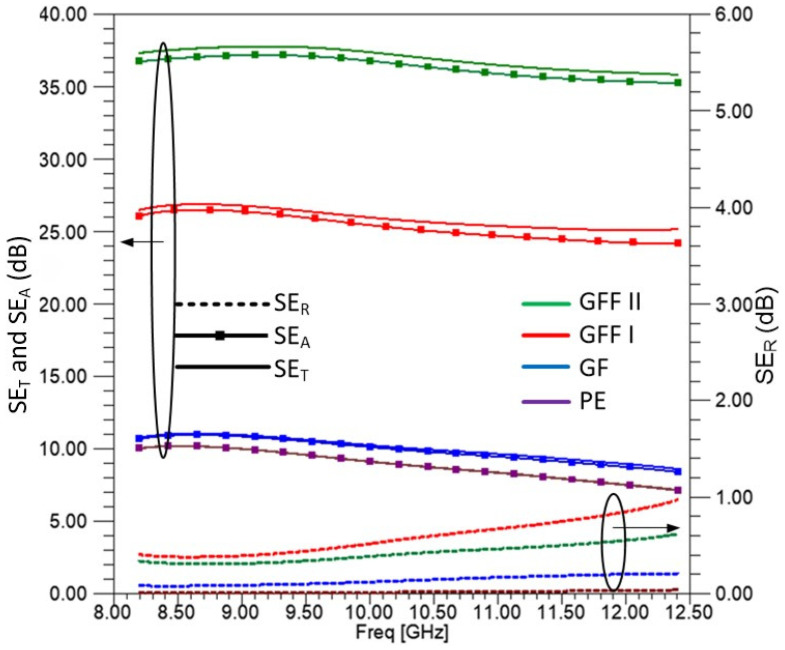
SE_R_, SE_A_, and SE_T_ for the samples of the materials (PE, GF, GFFI, and GFFII). Solid lines (-) refer to SE_T_, dashed lines (---) refer to SE_R_, and solid filled lines (−▪−) refer to SE_A_ for each of above materials.

In this section, we analyze the shielding effectiveness (SE) in decibels (dB) for all measured samples. Figure 16 presents the contributions to SE from reflection (SE_R_), absorption (SE_A_), and the total shielding effectiveness (SE_T_) at an incident angle of θ = 0 deg. As illustrated, SE_T_ is predominantly determined by SE_A_, with SE_R_ contributing minimally to the overall shielding performance. It is seen that for the SE_T_, an increase of 15 dB, from around 10 dB to 25 dB, is presented for the GFF I sample as compared to the GF and PE samples. An even higher increase of more than 10 dB, reaching 37 dB, is presented for the GFF II sample.

In Table 2, a comparison with other studies in the literature is carried out regarding carbon-based composite structures. The results for electromagnetic shielding effectiveness are shown for each composite structure according to its thickness and filler content. Nanomaterials are incorporated in various combinations to create carbon-based structures within different polymer matrices and filler content ratios. In this study, thin films are fabricated using multi-walled carbon nanotubes (MWCNTs) at 40 wt% combined with polyvinylpyrrolidone (PVP). Samples with one or two of such films are presented. As it is shown, films with a thickness of around 70 μm for the one-film sample and around of 140 μm for the two-film sample can reach a high EMI SE.

What distinguishes the present study from previously reported combinations is not only the relatively high MWCNT loading (40 wt%) but also the strategic simplicity, thin-film architecture, and scalability of the fabricated structures. Unlike many prior works that employed multilayered or hybrid composites with complex fabrication routes, our study utilizes a straightforward and reproducible process to create thin, flexible CNT-based films with competitive or even superior electromagnetic shielding performance. As shown in Table 2, while several earlier studies achieve a high shielding effectiveness (SE), these often require significantly thicker materials (e.g., 400–600 μm in [85,86,87]) or intricate combinations of nanofillers and polymer matrices.

In contrast, our MWCNT/PVP-based films achieve up to 38 dB EMI SE with only a 142 μm thickness, and 26 dB with just 71 μm, placing them among the most efficient in terms of EMI SE per unit thickness. This is accomplished using a single nanofiller system, avoiding additional processing complexity and cost. Furthermore, unlike earlier studies, we assessed the influence of stacking (one vs. two films) by investigating and comparing two distinct structural configurations with varying thicknesses and measurement distances, providing a more practical understanding of the material’s electromagnetic behavior.

These findings underscore the tunability and adaptability of our approach, as shielding performance can be enhanced through simple structural adjustments without altering the composition. This makes the proposed material highly promising for lightweight, compact, and flexible EMI shielding applications, especially in modern electronic devices where space, weight, and processability are critical design constraints. Altogether, these advantages emphasize the practical relevance and novelty of the present work compared to other combinations listed in Table 2.

### 3.7. Planar Periodic Structures

Electromagnetic designs featuring planar layers and periodic boundary conditions can be employed to simulate large surface areas. A variety of configurations can be created, comprising single-layer or multilayer structures, depending on the specific requirements of the application. Designs with successful shielding performance must lead to a minimized transmission coefficient. However, when designs with high absorption performance are considered, both reflection and transmission have to be minimized, as the absorption is a function of both S_11_ and S_21_ (see Equation (2)). Therefore, to maximize absorption, impedance matching between the layers and free space is essential. Achieving this requires a careful consideration of the intrinsic impedance of each layer. When the value of the impedance of each layer is close to the free space’s impedance, the reflection coefficient is close to zero, according to Equation (4). For this reason, the criterion of the decreasing microwave characteristic impedance law [94] is usually used to minimize reflections and achieve matching conditions.

**Figure 17:**
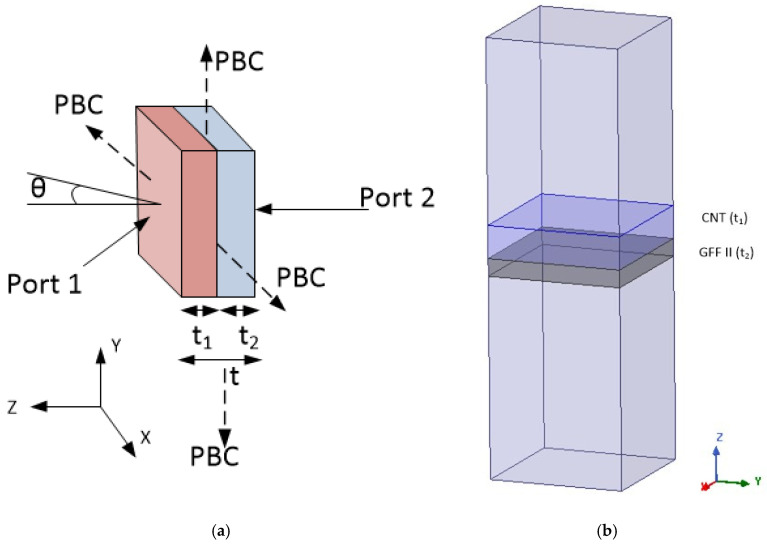
Unit cell description for the planar multilayer structure and incident angle θ in (**a**) schematic view and (**b**) in HFSS view.

To simulate large surfaces, a unit cell is typically designed with periodic boundary conditions (PBCs) applied to its side faces, as illustrated in Figure 17. The unit cell shown in Figure 17a is placed in a computational region box in HFSS simulation software, where the above periodic conditions are applied (Figure 17b). Since the one-layer structure designs presented in [68] do not seem to achieve high absorption independently of the material used, we examine here a two-layer design structure. The thickness of each layer in the unit cell is on the order of millimeters, while the transverse dimensions are considered infinite, rendering the structure electrically large. To simulate an incident plane wave, a Floquet port is used to excite the structure on both the top and bottom layers. TE and TM polarized plane waves are assumed to propagate either normally or at an oblique incident angle (θ) relative to the plane of the structure. The S parameters (S_11_ and S_21_) are then calculated to determine the reflection coefficient (RC) and transmission coefficient of the structure.

According to Section 3.5, where the S parameters from the samples were measured, it is seen that both GFF I and GFF II samples have a quite high S_11_ parameter. This results in high reflection. However, as shown in Figure 15b, S_21_ is very low, indicating minimal transmission. For this reason, we have chosen to place GFF II only on the bottom layer of the structure to prevent excessive reflection and reduce transmission. Any of the other materials can be applied to the top layer of the structure. We can use the material samples presented here or in [68]. According to [68], the CNT material sample, measured with the waveguide method, had the best matching conditions for maximum absorbance. Consequently, we designed a two-layer structure with CNT material from [68] for the top layer and GFF II for the bottom layer. In this design, the thickness of the bottom GFF II layer is kept constant at t_2_ = 1.65 mm. This thickness was previously used for the free space measurement process. An optimization analysis was performed for the top layer in order to minimize both S_11_ and S_21_. The thickness of the top layer can range from t_1_ = 2.5 mm to t_1_ = 3.5 mm, which results in maximum absorption corresponding to the optimal thickness of the top layer. The absorption, expressed as the Loss Factor, is presented here for TE incident waves, while similar results for TM waves are omitted for brevity.

**Figure 18:**
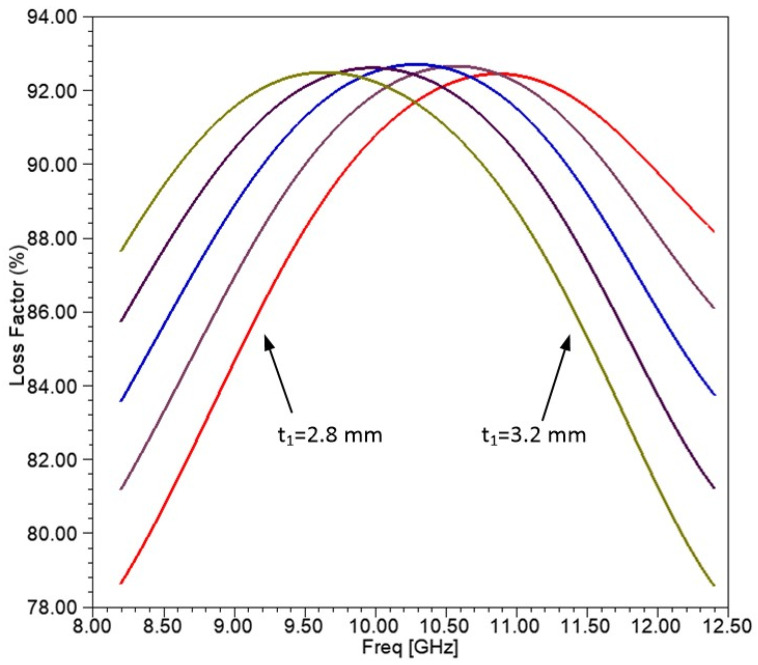
Loss Factor (%) for the planar structure for t_1_ = 2.8–3.2 mm and incident angle θ = 0 deg.

The optimum thickness derived from the optimization analysis for the top layer is around 3 mm. However, many manufacturing imperfections usually occur. Therefore, we assessed the robustness of the optimal results by analyzing deviations from the ideal value. To achieve this, we performed a parametric analysis where the thickness of the top layer is varied from t_1_ = 2.8 mm to t_1_ = 3.2 mm in increments of 0.1 mm. Figure 18 shows results for the absorption for all step values. As seen, absorption shows similar results for all thickness values. For higher thicknesses, the maximum absorption occurs at slightly lower frequencies.

This approach also enables a sensitivity evaluation, the fine-tuning of the absorption characteristics, and improved frequency selectivity. By precisely adjusting the parameters of the CNT layer, the absorption frequency can be shifted without significantly compromising the peak absorption performance. This level of tunability offers valuable flexibility for developing application-specific electromagnetic shielding solutions.

It is also well-established in the microwave literature that increasing the thickness of an absorbing layer shifts the frequency of maximum absorption toward lower values [95,96]. This behavior arises from the quarter-wavelength resonance condition, where phase cancellation due to destructive interference occurs at longer wavelengths—i.e., lower frequencies—as thickness increases. In multilayer absorbers composed of conductive or dielectric layers, this effect leads to a predictable shift in the resonant frequency. While the absorption magnitude primarily depends on impedance matching and intrinsic material losses, the position of the absorption peak is strongly influenced by layer thickness.

**Figure 19:**
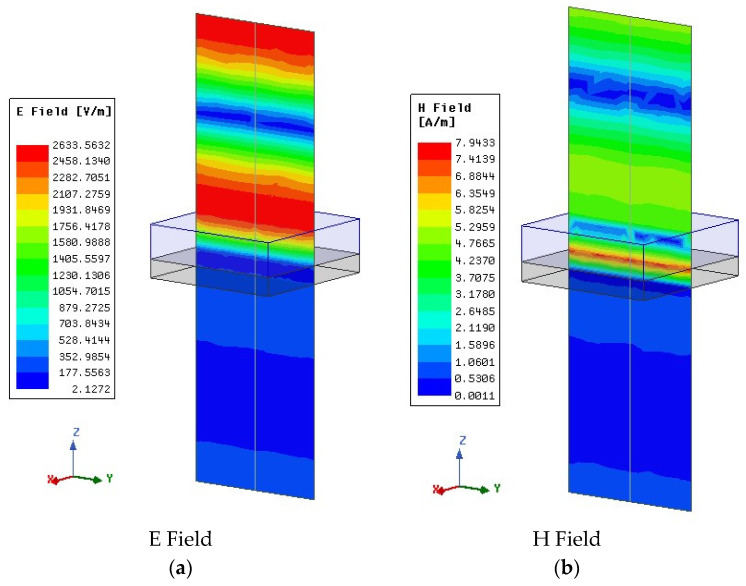
Magnitudes of the electric (E) (**a**) and magnetic (H) field (**b**) intensities for the planar multilayer structure.

In addition to absorption, the propagation of an EM wave through the planar multilayer structure is also examined. Figure 19a,b illustrates the electric and magnetic fields of an incident plane wave as they propagate perpendicular to the periodic planar structure. As it is shown, both fields are highly evanescent while penetrating the structure.

**Figure 20:**
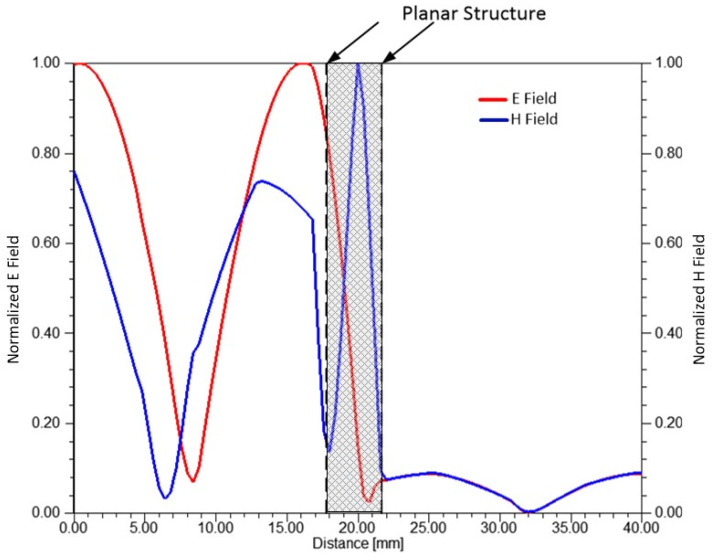
Normalized magnitudes of the E and H field intensities for the planar structure.

Figure 20 shows the normalized magnitudes of the E and H fields. The planar structure, represented by the unit cell, has a thickness of 4.65 mm and is positioned 17 mm from the edge of the region box in Figure 17b. As seen, both the E and H fields are significantly attenuated. Therefore, designs incorporating GFF materials appear promising for electromagnetic shielding and absorption applications.

Various nanomaterials with different weight percentages have been proposed for multilayered structures in the literature. In [44], nanopowder-based materials consisting of carbon nanotubes and various metals, including cobalt, silver, titanium, nickel, zinc, copper, iron, boron, bismuth, and hafnium, are incorporated into an epoxy polymer matrix. For the titanium-based material (TiC—titanium carbide), the CNT content is 2 wt%, whereas for all the other materials listed, the CNT content is 0.1 wt%. An optimization algorithm is used to design multilayer structures composed of the materials tested, which have thickness from 10 mm up to 52 mm, while minimum value of RC is around −18 dB. In [25], nanoconstructed multilayer absorbers are designed using a genetic algorithm. Single-walled carbon nanotubes (SWCNTs), multi-walled carbon nanotubes (MWCNTs), carbon nanofibers (CNFs), and fullerenes are incorporated at weight percentages of 1%, 3%, and 5% to form multilayer structures. The reflection and transmission coefficients, along with absorption expressed as the Loss Factor (%), are presented for various design configurations. The optimization algorithm determines the total thickness of structure of equal to around 11 mm. The structure achieves an RC around −5 dB and TC −32 dB. In [73], multilayer absorbing structures with known electromagnetic properties are fabricated using carbon-based nanomaterials such as CNTs, CNFs, PANI, and GNP. These structures have a minimum thickness of approximately 17 mm, with reflection and transmission coefficients around −5 dB and −22 dB, respectively. In a previous study [68], we presented a two-layer structure consisting of epoxy/MWCNTs (1 wt%) and GFF (MWCNTs 40 wt%/PVP). The measurements were based on waveguide experimental process. The above structure presented a high absorption percentage of 85–95% over the X-band frequency range, with a thickness of 5.7 mm. Here, using a much more thorough free space experimental procedure, we propose a thinner, two-layer design with equivalent a high absorption percentage of 80–93%. The total thickness of this two-layer structure is now 4.65 mm. The electrically large samples tested introduce the idea of covering large structures with the proposed layer.

## 4. Conclusions

Electromagnetic compatibility (EMC) is essential in aerospace systems, where electromagnetic interference (EMI) can compromise the functionality of critical sensors and onboard electronics, affecting safety and mission success. To mitigate such challenges, this study proposes and experimentally validates a novel class of electrically large, lightweight, and thin composite sheet structures for high-performance electromagnetic shielding and absorption in the X-band frequency range (8.2–12.4 GHz).

The proposed structures consist of thin (∼70 μm) freestanding multi-walled carbon nanotube (MWCNT) films embedded within a glass fiber polymer matrix. Two main configurations were investigated: one with a single CNT film at the mid-plane and another with two CNT films near the outer layers. The total thickness of the resulting structures remains under 2 mm, preserving a low weight while significantly enhancing electromagnetic performance.

Free space S parameter measurements using both rectangular and spot-focusing lens horn antennas, along with time-domain calibration techniques, demonstrated that the inclusion of CNT films substantially improved shielding and absorption behaviors. The two-film configuration achieved a transmission coefficient as low as −37 dB and a shielding effectiveness exceeding 35 dB. Furthermore, an optimized two-layer structure composed of CNT and GFF layers achieved a peak absorption of approximately 93% near 10 GHz, with a total thickness of just 4.65 mm.

Mechanical integrity was also preserved, as short-beam shear tests showed that placing the CNT films near the outer surfaces had no negative impact—and may even slightly enhance—the interlaminar shear strength (ILSS) of the composite.

The novelty of this work lies in the combination of a simple, scalable, water-based fabrication method for CNT films, the strategic embedding of CNT films into realistic composite laminates, and validation using full-scale free space measurement techniques. Together, these contributions offer a practical and effective solution for EMI mitigation in aerospace and advanced electronics, particularly in sensor-rich environments such as antenna enclosures, radar systems, navigation modules, and structural health monitoring systems.

These results demonstrate that the proposed CNT-based composites can provide high electromagnetic attenuation while maintaining structural performance and design flexibility, making them highly suitable for deployment in modern aerospace and other electromagnetically demanding applications.

## Figures and Tables

**Table 1:** Description of sheet material samples.

Sample	Description	Thickness
Polyethylene (PE)	Sample of polyethylene	t_1_ = 1.5 mm
Glass Fiber (GF)	Sample with glass fibers	t_1_ = 1.55 mm
Glass Fiber Film (GFF I)	Sample with glass fibers and one film of CNTs	t_2_ = 1.65 mm
Glass Fiber Film (GFF II)	Sample with glass fibers and two films of CNTs	t_3_ = 1.8 mm

**Table 2:** Comparison of the EMI shielding performance of composite film materials with existing literature references.

Material Samples	Filler Content	Thickness (μm)	Frequency (GHz)	EMI SE (dB)	Ref
PVB-PANI	60%-40%	268	8.2–12.4	13	[46]
PVB-PANI-FAC	60%-30%-10%	265	8.2–12.4	15	[46]
PVB-PANI-Co-FAC	60%-30%-10%	261	8.2–12.4	19	[46]
PVB-PANI-Ni-FAC	60%-30%-10%	259	8.2–12.4	23	[46]
SDBS: SWCNT/rGO/polyester fabrics/epoxy	1:99	600	8.2–12.4	40	[85]
SDBS: SWCNT/rGO/polyester fabrics/epoxy	1:99	600	8.2–12.4	40	[85]
PVDF/heterostructure/ MWCNT	Heterostructure (10 wt%)/MWCNT (3 wt%)	600	12–18	22–40	[86]
MWCNT/WPU	10.6% CNT	400	8.2–12.4	24.7	[87]
PANI/CNF/PVA, PANI/NFC/PVA	10%-40%	110	8.2–12.4	33	[88]
			2–4	31	[88]
GNP/EPDM	8 wt%	300	8.2–12.4	33	[89]
			12.4–18	35	
PANI-MnO_2_ nanorods	MnO_2_ nanorods 4:1	169	8.2–12.4	35	[90]
MWCNT/polyurethane	25% MWCNT	100	8.2–12.4	25	[91]
CNF/CF paper	12.8 wt%	167	0.5–1.0	24.6	[92]
PVB/Ni-Gr/SCF	70/25/5	200	8.2–12.4	32	[93]
**MWCNT/PVP/Glass Fiber (GFF I)**	**40 wt% MWCNT/PVP**	**71**	**8.2–12.4**	**26**	**This work**
**MWCNT/PVP/Glass Fiber (GFF II)**	**40 wt% MWCNT/PVP**	**142**	**8.2–12.4**	**38**	**This work**

## Data Availability

The original contributions presented in this study are included in the article. Further inquiries can be directed to the corresponding author.
